# The Golgi Ca^2+^ stores, and original contributions by Prof. Shao Bai Xue

**DOI:** 10.52601/bpr.2023.230015

**Published:** 2024-10-31

**Authors:** Zong Jie Cui

**Affiliations:** 1 College of Life Sciences, Beijing Normal University, Beijing 100875, China

**Keywords:** Golgi apparatus, Ca^2+^ stores, Secretory pathway Ca^2+^ ATPase (SPCA), Golgi anti-apoptotic protein (GAAP), Emetine

## Abstract

The Golgi apparatus serves as a distinct part of intracellular Ca^2+^ stores. Here, the early discovery by Professor Shao Bai Xue is reviewed, and the recent progress in the field is outlined. Golgi Ca^2+^ stores-related functional proteins, such as secretory pathway Ca^2+^ ATPases (SPCA1/2) and the Golgi-specific Ca^2+^ releasing channel Golgi anti-apoptotic protein (GAAP), as well as the recently defined Golgi-specific Ca^2+^ release agent emetine, collectively corroborate the concept of the Golgi apparatus as unique internal Ca^2+^ stores.

## INTRODUCTION

**Figure 1:**
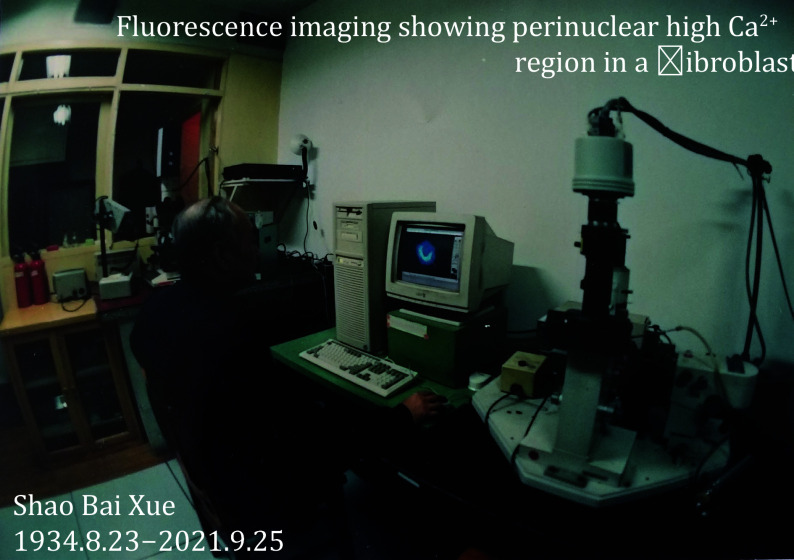
Professor Shao Bai Xue, demonstrating a single cell with a bright perinuclear fluorescent area. The photo was provided by the late Professor Shao Bai Xue and used in Cui ZJ, 2019. The lettering on the photo has been added by the present author

**Figure 2:**
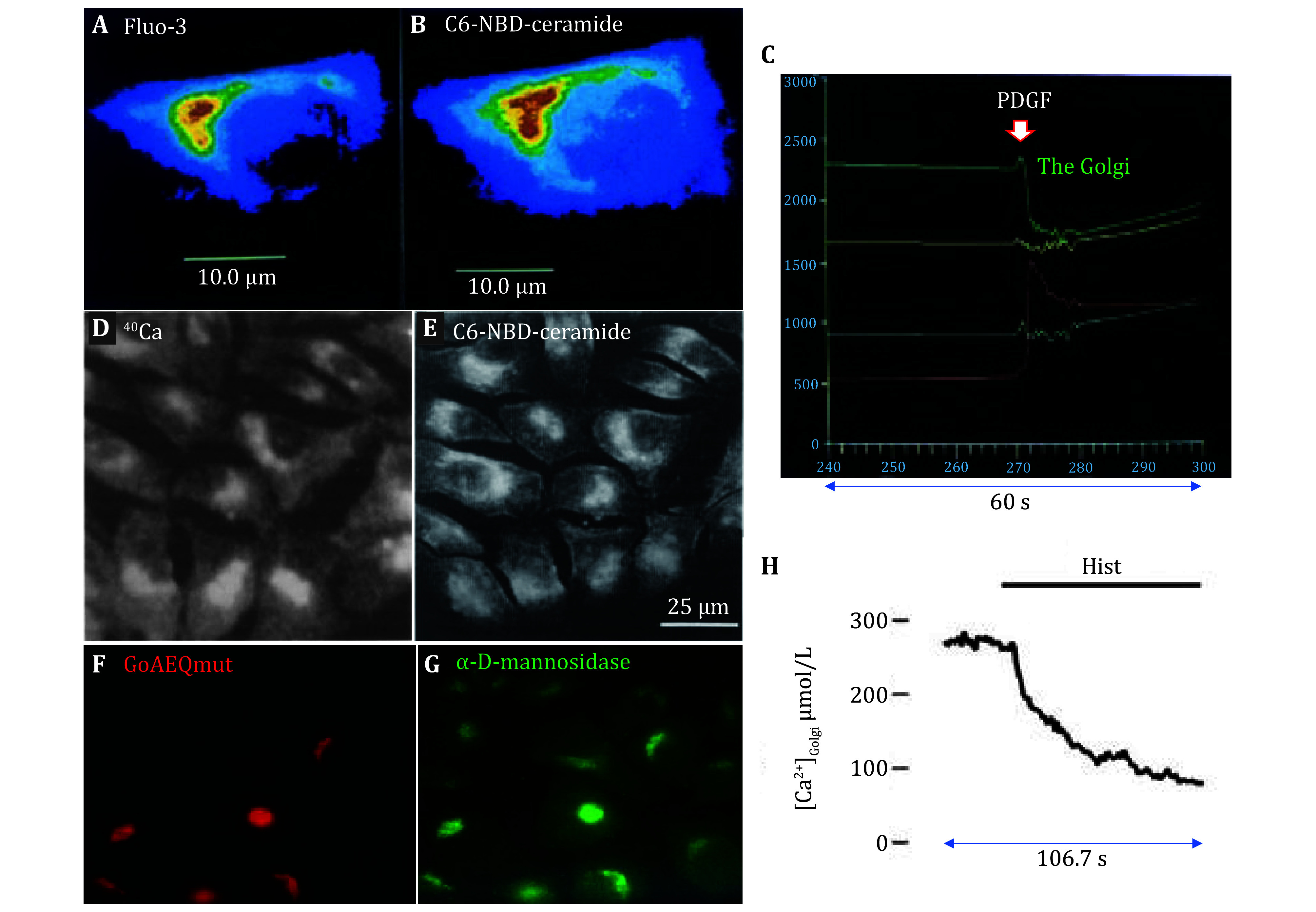
Perinuclear Golgi apparatus as Ca^2+^ stores. Ca^2+^ indicator Fluo-3 (**A**) and Golgi fluorescent probe C6-NBD-ceramide (**B**) colocalized in the perinuclear region in the same 2BS fibroblast cell. **C** PDGF (5 μg/mL) induced varied changes in Fluo-3 fluorescence in different regions in the same 2BS cell. Green: Golgi region; red: nucleus; yellow and blue: two regions of cytosol. The white arrow indicates the time of PDGF addition. Note the decrease in Golgi Ca^2+^ and increase in nuclear Ca^2+^. Panels A, B and C are adapted from Cui *et al*. (1995). **D**,**E** ^40^Ca distribution by total ion microscopy (**D**) and C6-NBD-ceramide fluorescent image by laser scanning confocal microscopy (**E**) in freeze-dried LLC-PK1 proximal tubular cells. Cells were exposed to a Ca^2+^-free medium for 5 min before cryogenic sample preparation. Panels D and E are adapted from Zha *et al.* (1995). **F**,**G** Immunocytochemistry of aequorin mutant GoAEQmut (**F**) and Golgi apparatus marker α‐D‐mannosidase II (**G**) in GoAEQmut-HeLa cells. **H** The Golgi luminal [Ca^2+^]_L_ decreased after stimulation with histamine 100 μmol/L in a transfected HeLa cell. Panels F, G and H are adapted from Pinton *et al*. (1998). Images in Panels F and G were taken with a 63× objective

The Golgi apparatus is strategically located in the protein transport and secretory pathway, being particularly prominent in secretory epithelia such as the pancreatic acinar cells. The Golgi apparatus, together with the endoplasmic reticulum (ER), mitochondria, lysosomes, and secretory granules, constitute distinct intracellular Ca^2+^ stores. This minireview outlines: (1) major works important in the establishment of the concept of the Golgi apparatus as intracellular Ca^2+^ stores, with a specific statement on the contributions by Professor Shao Bai Xue at Beijing Normal University (Fig. 1); (2) the current status of the field, with emphasis on functional proteins vital for the normal function of the Golgi apparatus as Ca^2+^ stores and on Golgi Ca^2+^ stores-specific agent emetine.

## EARLY EVIDENCE FOR THE PERINUCLEAR GOLGI AS A Ca^2+^ STORE

Early works about the Golgi apparatus as internal Ca^2+^ stores were performed on fibroblasts NIH3T3 by Shao Bai Xue of Beijing Normal University. Fibroblasts grown on coverslips were loaded sequentially with fluorescent Ca^2+^ indicator Fluo-3 (Fluo-3 AM, 10 µmol/L, 30 min), and fluorescent Golgi localizer C6-NBD-ceramide (10 min), and sequentially confocal imaged. Fluo-3 fluorescence was found concentrated at the perinucleus region, overlapping the Golgi indicator C6-NBD-ceramide in the same cells (Figs. 2A and 2B). This is possible because C6-NBD-ceramide is much brighter than Fluo-3, the CCD detection could be turned down (to the point that Fluo-3 fluorescence was no longer detected) after Fluo-3 imaging, before C6-NBD-ceramide loading and imaging (Xue *et al*. 1994). The overlapping of high Ca^2+^ region (Fluo-3 fluorescence) with Golgi localization (C6-NBD-ceramide fluorescence) was found conspicuous in interphase fibroblasts, but both concentration/localization (Fluo-3 and C6-NBD-ceramide) disappeared in metaphase when the Golgi apparatus disintegrated (Xue *et al*. 1994).

When Fluo-3-loaded interphase NIH3T3 cells were permeabilized with digitonin in Ca^2+^-free extracellular buffer, the decrease in Fluo-3 fluorescence in the Golgi region was found to be much smaller than in the non-Golgi regions (where near complete loss of Fluo-3 fluorescence is typically seen) (Table 1); it was proposed that the remaining Fluo-3 molecules in the Golgi region resided in the Golgi lumen (Xue *et al*. 1994).

**Table 1:** Fluo-3 fluorescence is retained at the perinulcear Golgi apparatus after plasma membrane permeabilization with digitonin (Xue *et al*. 1994)

	Fluo-3 Fluorescence (*n* = 30 NIH3T3 cells)	
	Golgi regions	Non-Golgi region of cytosol
Control	143.46	64.68
Digitonin (10 μmol/L, 1 min)	92.75	16.07
Fluorescence retention (%)	64.7%	24.8%

Sequential Fluo-3 and C6-NBD-ceramide loading and imaging in human lung fibroblast 2B cells grown on coverslips similarly revealed over-lapping of Fluo-3 and C6-NBD-ceramide fluorescence, *i*.*e*., high Ca^2+^ region was actually the Golgi apparatus (Figs. 2A and 2B). Stimulation of human lung embryonic fibroblast 2BS cells with platelet-derived growth factor (PDGF, 5 µg/mL) in Ca^2+^-free extracellular medium revealed in the same cell, of instantaneous decrease of luminal Ca^2+^ in the Golgi, and simultaneous increase of nuclear Ca^2+^ (Fig. 2C), indicating nuclear uptake of Ca^2+^ released from the Golgi apparatus; these initial parallel transients were followed by continued Ca^2+^ increases in the Golgi, nucleus, and cytosol (Cui *et al*. 1995). The later phase of Ca^2+^ increases after PDGF stimulation was proposed to be due to ER stores Ca^2+^ release, leading to increases in cytosolic, nuclear Ca^2+^, and eventual Ca^2+^ re-uptake by the Golgi.

The sarcoendoplasmic reticulum Ca^2+^-ATPase (SERCA) inhibitor thapsigargin (Tg, 200 ng/mL) when added to Fluo-3-loaded 2BS cells, was found to induce similarly sequential Ca^2+^ increases in the Golgi apparatus, the nucleus, and the cytosol (Cui *et al*. 1995).

The above confocal imaging data from mouse embryonic fibroblasts NIH3T3 cells, human lung embryonic fibroblast 2BS cells, and mouse embryonic fibroblast C3H10T1/2 cells (Xue *et al*. 1994; Cui *et al*. 1995), provided convincing morphological and functional support for the existence of the Golgi Ca^2+^ stores. These works were published in 1994 and 1995 (on 1 June 1994 and 1 December 1995) respectively (Xue *et al*. 1994; Cui *et al*. 1995), around the same time as work to suggest that the Golgi apparatus was internal Ca^2+^ stores in porcine renal proximal tubular epithelial cells (Zha *et al*. 1995) (published on 1 May 1995).

The Morrison laboratory reported that in Calcium Green-loaded porcine renal proximal tubular epithelial cell LLC-PK1, after disruption of the Golgi apparatus with brefeldin A (BFA), vasopressin-induced cytosolic calcium increases were much reduced (Zha *et al*. 1995). If these epithelial cells were freeze-dried for total Ca ion microscopy, and confocal imaged after loading with fluorescent Golgi indicator C_6_-NBD-ceramide, the spatial distribution of ^40^Ca and the fluorescent Golgi indicator C_6_-NBD-ceramide were found similar in distribution at the perinuclear region (Figs. 2D and 2E). In addition, 1 min after stimulation with vasopressin, total Ca in the perinuclear Golgi region was found to start to decrease significantly; brefeldin-A treatment led to similar reduction over a more extended time frame (10–30 min) the total Ca (gauged by total ion microscopy) in the perinuclear Golgi region. These authors similarly proposed that the Golgi apparatus is part of intracellular Ca^2+^ stores (Zha *et al*. 1995).

More elaborate experiments were carried out a few years later, after the above works had been published. For example, the photoprotein aequorin was target-expressed to the Golgi in HeLa cells, after tagging at the aequorin N-terminus with both sialyltransferase (ST) and HA1 (to obtain ST-HA1-GoAEQmut^Asp119Ala^). It was confirmed that in positive expressing GoAEQmut-HeLa cells, the GoAEQmut co-localized with the Golgi resident enzyme α‐D‐mannosidase (Pinton *et al*. 1998) (Figs. 2F and 2G). Histamine stimulation of such GoAEQmut-HeLa cells led to rapid and extensive emptying (within 1.5 min) of the Golgi luminal calcium (Pinton *et al*. 1998) (Fig. 2H). Further, overexpression of the Golgi luminal Ca^2+^-binding protein CALNUC was found to increase the total size of intracellular Ca^2+^ stores (gauged by total ^45^Ca^2+^ uptake per 10^6^ HeLa cells) (Lin *et al*. 1999). Works done around the same time with the yeast similarly suggested that the Golgi was an intracellular Ca^2+^ store (Miseta *et al*. 1999).

The Xue group used the fluorescent Ca^2+^ indicator Fluo-3, with a *K*_d_ of 390 nmol/L (https://www.aatbio.com/catalog/calcium-indicators#Fluo3andFluo4), which would be useful for measurement of Ca^2+^ concentrations from 39 nmol/L to 3.9 µmol/L (0.1 to 10 times of *K*_d_) (Xue *et al*. 1994; Cui *et al*. 1995). Whereas the Morrison group used Calcium Green, with a *K*_d_ of 190 nmol/L (https://www.aatbio.com/catalog/calcium-indicators#Fluo3andFluo4) (suitable Ca^2+^ concentration range to measure is from 19 nmol/L to 1.9 µmol/L) (Zha *et al*. 1995). To examine the luminal Ca^2+^ concentration in the Golgi apparatus, probes with lower affinities (higher *K*_d_ values) would be more appropriate. Such Ca^2+^ store-suitable fluorescent Ca^2+^ probes have all appeared subsequently, both in the form of synthetic fluorescent chemical probes and designer genetically encoded fluorescent protein Ca^2+^ indicators.

The low affinity genetically encoded red fluorescent protein Ca^2+^ indicator LAR-GECO1 (*K*_d_ = 24 µmol/L), for example, when expressed in the ER or Golgi in HEK293 cells, and simultaneously loaded with synthetic fluorescent probe Fluo-8 (*K*_d_ = 389 nmol/L, https://www.aatbio.com/catalog/calcium-indicators#Fluo3andFluo4), could measure Ca^2+^ stores luminal Ca^2+^ and cytosolic Ca^2+^ simultaneously (Konieczny *et al*. 2017; Wu *et al*. 2014).

The Golgi is composed of *cis*-, medial and *trans*-Golgi. Morphological heterogeneity corresponds to the differences in the distribution of subregional specific enzymes therefore differences in Golgi function (Pizzo *et al*. 2011; Aulestia *et al*. 2015; Li and Wang 2022).

The genetically encoded fluorescent protein Ca^2+^ indicator Cameleon D1cpv, when tagged to the N-terminal sequence of 32 residues of Golgi resident enzyme 1,6 N-acetyl glucosaminyl transferase, could reveal that Ca^2+^ uptake by the medial-Golgi was powered by the sarcoendoplasmic reticulum Ca^2+^-ATPase (SERCA) and secretory pathway Ca^2+^-ATPase (SPCA). IP_3_ receptor or ryanodine receptor (RyR) activation triggered Golgi Ca^2+^ release. The medial-Golgi luminal Ca^2+^ concentration was measured and found to be higher than the *trans*-Golgi, but lower than in the ER (Wong *et al*. 2013). These data indicate that despite the continuous flow of both membrane and luminal content within the Golgi apparatus, each subregion maintains its own Ca^2+^ homeostasis (Wong *et al*. 2013).

Aequorin specifically target-expressed in the *cis*-, medial- and *trans*-Golgi could be used to measure and compare luminal Ca^2+^ concentration and total size of the Ca^2+^ stores both in the ER and Golgi. The luminal Ca^2+^ concentration ([Ca^2+^]_L_) in *cis*-Golgi was found to be about 150–300 μmol/L, where Ca^2+^ is taken up from the cytosol mainly by sarcoplasmic reticulum Ca^2+^-ATPase (SERCA) (Aulestia *et al*. 2015). The *trans*-Golgi is further divided into two regions: one region with secretory pathway Ca^2+^-ATPase 1 (SPCA-1) activity, and the second region with no SPCA-1 activity, but with SERCA activity. The two regions/compartments are separate with a determined diffusion barrier (Aulestia *et al*. 2015). IP_3_ could stimulate only one region of the Golgi compartments to release Ca^2+^, but could not mobilize Ca^2+^ from the more distant region (Aulestia *et al*. 2015). Caffeine releases Ca^2+^ from all regions of the Golgi apparatus, but nicotinic acid dinucleotide phosphate (NAADP) or cADP ribose (cADPR) could not (Aulestia *et al*. 2015).

The luminal Ca^2+^ concentration [Ca^2+^]_L_ decreases gradually, starting from the nuclear membrane, to the ER. ER [Ca^2+^]_L_ could be up to 500 µmol/L, *cis*-Golgi 250 µmol/L, medial-Golgi 190 µmol/L, and *trans*-Golgi 130 µmol/L, [Ca^2+^]_L_ in secretory granules is only about 80 µmol/L (Pizzo *et al*. 2011).

The Golgi apparatus in cardiomyocytes was found to be the source of continued increases in cytosolic Ca^2+^ in the perinuclear region. Ca^2+^ release from the Golgi apparatus was found not affected by depletion of SR Ca^2+^ stores; after the Golgi apparatus was disrupted, Ca^2+^ release from the Golgi stores disappeared, but this would not affect Ca^2+^ release from the SR; data indicated that SR and Golgi Ca^2+^ stores are completely independent both morphologically and functionally (Yang *et al*. 2015). The Golgi Ca^2+^ stores function was found independent of the sarcoplasmic reticulum and global Ca^2+^ transient-induced cardiomyocyte contraction (Yang *et al*. 2015).

The Golgi apparatus takes up Ca^2+^ by sarco-endoplasmic reticulum Ca^2+^ ATPase (SERCA) and secretory pathway Ca^2+^ ATPase1 (SPCA1). Ca^2+^ will be released from the Golgi after activation of the IP_3_R. Expression of a genetically encoded Ca^2+^ indicator based on fluorescence resonance energy transfer (FRET) in *trans*-Golgi revealed that *trans*-Golgi was not responsive to IP_3_ stimulation, and took up Ca^2+^ via SPCA1 only (Pizzo *et al*. 2010). SERCA and IP_3_R were found expressed in *cis*- and medial-Golgi (Pizzo *et al*. 2010). Knockdown by small interfering RNA (RNAi) of SPCA1 expression was found to result in: (1) decreased [Ca^2+^]_L_ in *trans*-Golgi, (2) major changes in secretory pathway protein transport, and (3) disruption of the Golgi apparatus (Pizzo *et al*. 2010). These data indicated that SPCA1 plays an important role in Golgi structure and function.

## THE SECRETORY PATHWAY Ca^2+^-ATPASE IN THE GOLGI APPARATUS

In multiple cell types, cytosolic Ca^2+^ is taken up not only by P2A type ATPase of the sarco-endoplasmic reticulum Ca^2+^-ATPase (SERCA) and P2B type ATPase of the plasma membrane Ca^2+^-ATPase (PMCA), but also by P2A type ATPase of the secretory pathway Ca^2+^-ATPase (SPCA) expressed in the Golgi apparatus/secretory pathway.

The human genome contains two *SPCA* genes, encoding the ATPases SPCA1 and SPCA2. The two terminals of SPCA1,2 proteins have been found to interact with plasma membrane Ca^2+^ channel ORAI1, to mediate stores-independent (*i*.*e*., STIM1-independent) Ca^2+^ entry (SICE) (Feng *et al*. 2010; Smaardijk *et al*. 2018). A dysfunctional SPCA could lead to multiple human diseases (Chen *et al*. 2020).

SPCA1/2 transports Ca^2+^ and Mn^2+^ into the Golgi apparatus. SPCA2 was found to activate ORAI independent of STIM1, to mediate constitutive stores-independent Ca^2+^ entry (SICE) in lactating mammary glandular epithelial cells (Smaardijk *et al*. 2018). Similar to SPCA2, overexpressed SPCA1 was also found to induce STIM1-independent, but ORAI-dependent Ca^2+^ influx. This would lead to increases in cytosolic Ca^2+^ and non-ER Ca^2+^ stores, suggesting a functional coupling between Orai1 and SPCA1 (Smaardijk *et al*. 2018). Importantly, Orai1 and SPCA1a were found to colocalize at the plasma membrane (Smaardijk *et al*. 2018).

In patients with autosomal dominant Hailey-Hailey disease (HHD), persistent skin blisters and erosions are normally found. The Golgi-specific Ca^2+^-ATPase in Hailey-Hailey disease patient ATP2C1 shows multiple point mutations, leading to unbalanced Ca^2+^ homeostasis. Compared to healthy subjects, the basal Ca^2+^ concentration in keratinocytes of HHD patients was found to be much reduced (Hu *et al*. 2000).

Loss-of-function mutations of SPCA1 lead to dysfunction of SPCA1a/Orai1 coupling, as seen in Hailey-Hailey disease (HHD) (Smaardijk *et al*. 2018). HHD-related mutation of SPCA1a leads to dysfunction of Ca^2+^ transport and Orai1activation, and dysregulated Ca^2+^ content in non-ER calcium stores. Functional coupling of SPCA1 to Orai1 increases cytosolic and Ca^2+^ stores [Ca^2+^]_L_, this novel mechanism of Ca^2+^ stores-independent Ca^2+^ entry is affected in HHD (Smaardijk *et al*. 2018).

**Figure 3:**
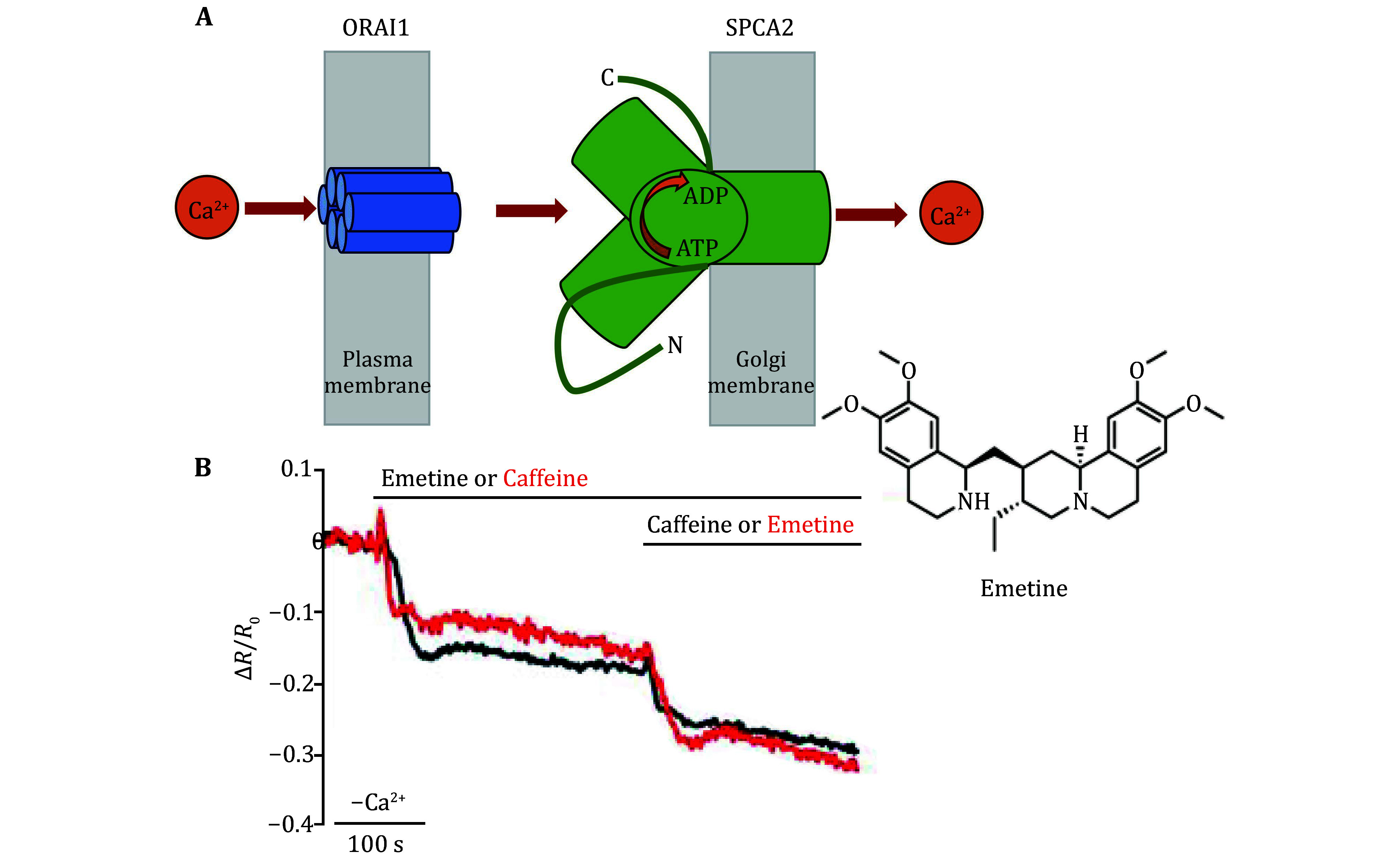
Ca^2+^ handling in the Golgi apparatus. **A** The N- and C-terminals of SPCA2 located across the membrane of the Golgi apparatus may interact directly with plasma membrane ORAI1 for Ca^2+^ entry and refilling of the Golgi apparatus. Redrawn after Smaardijk *et al.* (2017). **B** The trans-Golgi [Ca^2+^]_L_ in HL-1 mouse cardiac cells is released by treatment with emetine. Mouse HL-1 heart cells were transfected with fluorescent Ca^2+^ indicator GoD1cpv and maintained in a Ca^2+^-free medium. Changes in Δ*R*/*R*o ratio for cells that were stimulated with caffeine and then emetine (red trace), or in reverse order (black trace), are shown. Nuclear and SR luminal Ca^2+^ concentrations are affected by caffeine, but not by treatment with emetine. Note the structure of emetine at the top right corner of Panel B. Adapted from Gallegos-Gómez *et al.* (2018)

SPCA2 N- and C-terminals function to activate ORAI1 and Ca^2+^ transport to the Golgi/secretory pathway; truncation of N- and C-terminals will confirm such effect on SICE and Ca^2+^ transport (Smaardijk *et al*. 2017) (Fig. 3A). C-terminal truncation was found to decrease SICE, SPCA2 activity, and affect SPCA2 location; whereas N-terminal truncation affected SPCA2 localization, resulting in SPCA2 inactivation, but SICE remained quite normal. Overexpression of SPCA2 increased the capacity of non-ER Ca^2+^ stores, the store’s capacity was dependent upon the activities of both Orai1 and SPCA2 (Smaardijk *et al*. 2017). Orai1-mediated Ca^2+^ influx and SPCA2-mediated Ca^2+^ uptake by Golgi/secretory pathway are coupled in the same subcellular microdomain. This Ca^2+^ channel/ Ca^2+^ pump complex efficiently transfers Ca^2+^ into the secretory pathway, to regulate the secretion of SPCA2-expressing secretory cells such as lactating mammary glands (Smaardijk *et al*. 2017).

Different from its role to increase Golgi Ca^2+^ stores, increased SPCA2 expression stimulates Ca^2+^ influx, this is dependent upon Ca^2+^ stores-operated Ca^2+^ channel Orai1. However, SPCA2-Orai1 coupling is independent of ER Ca^2+^ stores or associated Ca^2+^ sensors STIM1,2, and independent of SPCA2 Ca^2+^- ATPase activity (Feng *et al*. 2010).

SPCA1 pumps cytosolic Ca^2+^/Mn^2+^ into the Golgi. Cryo-EM studies obtained human hSPCA1a structures at 3.1–3.3-Å resolution, both in the Ca^2+^/Mn^2+^-bound (E1-ATP) and divalent cation-free phosphorylated (E2P) states (Chen *et al*. 2023). From such structures it is obvious that Ca^2+^ and Mn^2+^ share the same cation binding pocket, but with different coordination bonds in the TM domains; this binding pocket corresponds to the second Ca^2+^ binding site in SERCA. During E1-ATP to E2P conversion, the binding domain recombination of SPCA1a is very similar to SERCA. SPCA1a structure reveals both spatial and positional flexibility of TM2 and TM6, likely indicating wider cation selectivity (Chen *et al*. 2023). In fact, in a more recent work, cryo-EM elucidation of a series of six intermediate state structures, including the long-sought-after CaE2P state (of P-type 2A ATPases), of the human SPCA1, nearly completed the atomic depiction of the Ca^2+^ entry and release cycle and mechanism (Wu *et al*. 2023).

Recently a series of five 1,3-thiazole compounds have been reported to have specific inhibitory activity towards hSPCA1. Compound 1 showed complete inhibition with submicromolar IC50 (0.8 µmol/L) towards hSPCA1, incomplete inhibition with IC50 of 2.6 and 2.7 µmol/L towards hSPCA2 and rSERCA respectively, but was ineffective for inhibition towards Na,K-ATPase or H,K-ATPase (Yamamoto-Hijikata *et al*. 2022). Complete SPCA1 inhibition by Compound 1 was achieved at 10 µmol/L, but at 10 µmol/L Na,K-ATPase or H,K-ATPase were inhibited only 47% and 37% respectively (Yamamoto-Hijikata *et al*. 2022).

The Golgi apparatus-located transmembrane Ca^2+^/Mn^2+^ exchanger/antiporter TMEM165, may work in either direction, to functionally interact with SPCA1 (Li and Wang 2022).

There are at least three different Ca^2+^-binding/buffering proteins inside the Golgi: Cab45, P54/NEFA and CALNUC (nucleobindin) (Lin *et al*. 1999; Gilon *et al*. 2014; Prins *et al*. 2011). Cab45 is the first member of the CREC family of proteins, the CREC acronym being taken from the first alphabet of each member protein: Cab45, reticulocalbin1, ERC-55 and calumenin (Wang *et al*. 2019). The Ca^2+^ ions conjugated by these proteins inside the Golgi are released into the cytosol via Ca^2+^-permeant cation channels. Such Ca^2+^ channels might include Golgi anti-apoptotic protein (GAAP).

## THE GOLGI Ca^2+^-RELEASING CHANNEL

Golgi anti-apoptotic protein (GAAP), a transmembrane protein, also named transmembrane Bax inhibitor-1 motif-containing 4 (TMBIM4), or Lifeguard 4 (Lfg4), is conserved in eukaryotes, prokaryotic cells and orthopox virus (Gubser *et al*. 2007) (Carrara *et al*. 2015). Eukaryotic GAAP regulates the content of intracellular Ca^2+^ stores. Purified camelpox virus GAAP (vGAAP), or the human homologue Bax inhibitor 1, after reconstitution in artificial lipid bilayers, form voltage-gated cation channels which open spontaneously (Carrara *et al*. 2015). Point mutated vGAAP shows changed single channel conductance (E207Q, D219N) or ion selectivity (E207Q) (Carrara *et al*. 2015). Residues Glu(E)207 and Asp(D)219 form part of the ion conductance pore (Carrara *et al*. 2015).

vGAAP after oligomerization forms Ca^2+^ channels across the Golgi membrane (Carrara *et al*. 2017). Oligomerization of the human and virus GAAP, as well as bacterial isoform BsYetJ, is regulated by pH (Carrara *et al*. 2015; Zhang *et al*. 2021). TMBIM1-4 are all expressed in the Golgi (Zhang *et al*. 2021). Interestingly, lysosomal Ca^2+^ channel TMBIM1 is regulated by pH (Pihán *et al*. 2021), and ER Ca^2+^ channel TMBIM6 is modulated by the lipid environment (Lan *et al*. 2023).

Unlike IP_3_R or RyR, which are activated by IP_3_ and caffeine respectively, no endogenous agonist is yet known to activate GAAP.

The plant-derived emetine has been found to release Ca^2+^ from the Golgi. In both HeLa cells and atrial myocyte HL-1, the emetic, anti-amoebic, and anti-SARS-CoV-2 agent, releases Ca^2+^ specifically from the Golgi Ca^2+^ stores (Gallegos-Gómez *et al*. 2018) (Fig. 3B). The *cis*-Golgi continuously acquires Ca^2+^ from the extracellular fluid or from the ER, to refill Ca^2+^ stores for release into the cytosol. Emetine-sensitive Ca^2+^ mobilization mechanism is completely different from the two known mechanisms for Ca^2+^ mobilization of IP_3_R and RyR (Gallegos-Gómez *et al*. 2018). Emetine-specific Ca^2+^ release from the Golgi is not due to inhibition of SPCA, but is likely due to activation of a Ca^2+^-permeant channel, and whether this is related to GAAP will need further studies (Gallegos-Gómez *et al*. 2018).

The human hGAAP not only regulates Golgi Ca^2+^ stores, but it is also required for cell survival, it is anti-apoptotic, and promotes cell adhesion and migration. Point mutation of camelpox virus vGAAP at Glu207 or Glu178 reduces vGAAP effect on cell migration and adhesion, but does not affect its anti-apoptotic function (Carrara *et al*. 2015). Asp219 mutation abolishes the anti-apoptotic effect of GAAP, but has no effect on cell migration or adhesion (Carrara *et al*. 2015). Overexpressed human hGAAP promotes 3-dimensional matrix protease-dependent cell infiltration via reactive oxygen species; overexpressed hGAAP promotes mitochondrial respiration (Almeida *et al*. 2020).

## CONCLUSION AND PERSPECTIVES

From early confocal data with Ca^2+^ indicator fluorescence concentrated in perinuclear Golgi, to Golgi-localized expression of fluorescent protein Ca^2+^ indicators of low affinity, to identification of secretory pathway Ca^2+^-ATPase (SPCA), elucidation of Ca^2+^-permeant channels of GAAP, and recent discovery of emetine to specifically release Ca^2+^ from the Golgi, the Ca^2+^ stores function of the Golgi apparatus is becoming clearer than ever before.

Strategically located in the secretory pathway, the Golgi Ca^2+^ stores, together with Ca^2+^ stores of ER and secretory granules, show distinct patterns of expression in Ca^2+^-handling proteins. However, the detailed maturation process of G protein-coupled receptors in transit at the Golgi, and possible correlation to their Ca^2+^ store function, are not fully understood. We have recently found that a certain A-class receptor, the cholecystokinin 1 receptor (CCK1R), could be permanently activated in photodynamic action at targeted subcellar organelles (Li and Cui 2022), this approach might help to fully elucidate the maturation process of GPCR in the secretory pathway, and at the Golgi apparatus in particular. Transmembrane proteins at the Golgi apparatus not involved directly in Ca^2+^ handling might also affect the Ca^2+^ store function. In vascular smooth muscle cells, for example, the vesicular chloride transporter ClC-6 is highly expressed in the Golgi apparatus; reduced Golgi expression of ClC-6 is known to decrease the capacity of the Golgi Ca^2+^ stores (Klemens *et al*. 2021). It should be noted that in different cell types, the function of the Golgi might vary. Just like the perinuclear localization of the Golgi in fibroblasts, as discussed in this minireview, in other types of cells, such as the polarized secretory epithelial cells - the pancreatic acinar cells in particular, the Golgi is located at the apical side of the mitochondria zone, but at the basal side of the zymogen granules (in between the ER and zymogen granule region), with a distinct distance from the nucleus (Dolman *et al*. 2005).

A varied version of this account has been published in Chinese (Cui 2023), geared more for the Chinese-speaking scientific community.

## Conflict of interest

Zong Jie Cui declare that they have no conflict of interest.
